# Age- and gender-specific acute poisoning with drugs and medications affecting nervous system

**DOI:** 10.1186/s40360-024-00759-1

**Published:** 2024-07-01

**Authors:** Bita Mesgarpour, Shabnam Faridfar, Mahya Rezaei, Akbar Abdollahiasl, Shahin Shadnia, Arezou Mahdavinejad, Mohammad Abdollahi

**Affiliations:** 1 Cochrane Iran Associate Centre, National Institute for Medical Research Development (NIMAD), Tehran, Iran; 2 Department of Epidemiology, School of Public Health, Iran University of Medical Sciences, Tehran, 1449614535 Iran; 3 Department of Pharmacoeconomics and Pharmaceutical Administration, Faculty of Pharmacy and Pharmaceutical Sciences Research Center (PSRC), Tehran University of Medical Sciences, Tehran, Iran; 4 Department of Clinical Toxicology, Toxicological Research Center, Excellence Center of Clinical Toxicology, Loghman Hakim Hospital, Shahid Beheshti University of Medical Sciences, Tehran, Iran; 5 Department of Toxicology and Pharmacology, Faculty of Pharmacy and Pharmaceutical Sciences Research Center (PSRC), Tehran University of Medical Sciences, Tehran, 1417614411 Iran

**Keywords:** Acute poisoning, Suicide, Epidemiology, Opium, Methadone, Iran

## Abstract

**Background:**

We investigated acute poisonings resulting from medications affecting the nervous system and illicit substances at Loghman Hakim Hospital in Tehran.

**Methods:**

We retrospectively reviewed patient records at Iran’s largest tertiary toxicology referral center between January 2010 and December 2015. We analyzed the prevalence, trend, age and gender distribution of acute poisoning caused by nervous system agents.

**Results:**

The present study included 16,657 (57.27%) males and 12,426 (42.73%) females, resulting in 29,083 patients. The median age of men and women was 29 and 26 years, respectively (*p* < 0.0001). There were 12,071 (72.47%) men and 10,326 (83.10%) women under the age of 40 (*p* < 0.001). Most cases were intentional (69.38% in men and 79.00% in women, *p* < 0.001) and 44.10% had a history of poisoning. The proportions of men and women varied significantly between different age groups and nervous system agents. For women, the most common agent was alprazolam, whereas for men, methadone. The overall trend of acute poisoning with drug used in addictive disorders, opioids and alcohol was increasing but decreasing with benzodiazepines and antidepressants. Acute poisoning by nervous system agents led to more deaths in men (1.95% vs. 0.56%; *p* < 0.001).

**Conclusions:**

Methadone intoxication was common especially among young men and most of these intoxications were intentional. Women and men aged 20–29 most frequently suffer poisoning from alprazolam and clonazepam, respectively. Women over 60 and men over 30 used opium. Illicit drugs caused more than half of the deaths, and opium dominated. This study may create awareness and develop educational and preventive gender and age-specific local programs.

**Supplementary Information:**

The online version contains supplementary material available at 10.1186/s40360-024-00759-1.

## Background

Acute poisoning can result from a single or multiple exposures to a toxic substance within a short period, either intentionally or unintentionally. In addition to causing significant morbidity and mortality, it also poses a significant challenge to health care systems globally [1–4]. Various factors influence poisoning patterns in a country, including geography and climate, social and economic factors, culture, and religious beliefs of the society [5]. According to the World Health Organization (WHO), approximately 2–3 million unintentional acute poisoning cases occur worldwide yearly. In most cases, poisoning occurs in developing countries due to inadequate resources and a lack of regulation, contributing to poisoning being an increasing prevalent condition [6–8].

Various substances can cause acute poisoning, including industrial chemicals, household products, medications, illicit drugs, and environmental pollutants [9–11]. Undoubtedly, drugs and medications that affect the nervous system are among the most significant subsets of toxic exposures. These drugs have profound effects on both the central and peripheral nervous systems, leading to a range of neurological symptoms and complications [12, 13]. It has been found that opioids, benzodiazepines, and antidepressants are the most common causes of poisoning in the United States [14]. There is also evidence that antipsychotics, antidepressants, and hypnotics/ sedatives are commonly implicated in cases of poisoning that affect the nervous system [15].

Although acute poisoning affects people of all ages and genders, certain populations are particularly vulnerable to specific toxins, highlighting the need for age- and gender-specific research and interventions [16, 17]. For instance, older adults may be more susceptible to poisoning with benzodiazepines and opioid analgesics, whereas younger adults may be more at risk of poisoning with illicit drugs and prescription stimulants. Additionally, the risk of medication poisoning may differ according to gender due to differences in drug metabolism, hormonal factors, and social determinants of health [18–22]. As reported by Wightman et al., in accidental opioid-involved overdose deaths, women were more likely to be exposed to benzodiazepines, antipsychotics, and antidepressants than men [23]. In terms of gender differences, several studies have shown that males are more likely than females to become involved in acute drug poisoning. Intentional self-harm is more common in women, while accidental overdoses and substance abuse-related poisonings are more prevalent in men [24–27].

The nature and patterns of drug and medication abuse have changed with the introduction of new drugs and medications on the market. For this reason, it is imperative that we understand the epidemiology of acute poisoning and the factors contributing to its occurrence to develop effective prevention strategies. In this study, our primary objective was to study the age and gender-based disparity in cases of acute poisoning caused by substances affecting the nervous system. Our secondary objective was to examine the pattern and types of poisonous agents and the associated mortality.

## Methods

We retrospectively reviewed all cases of acute poisonings with substances acting on the nervous system (Anatomical Therapeutic Chemical (ATC) Code N) admitted to the Loghman-Hakim Hospital Poison Center (LHHPC) from 1st January 2010 to 22nd December 2015. LHHPC is a teaching reference hospital for poisoning in Tehran, Iran.

The cases assigned with diagnosis code T40: Poisoning by narcotics and psycholeptics (hallucinogens), T42: Poisoning by antiepileptics, hypnotics-sedatives, and antiparkinsonian agents, and T43: Poisoning by psychotropic drugs, not elsewhere classified based on the 10th International Statistical Classification of Diseases and related health problems (ICD-10) coding system were retrieved. We did not include code T51 (toxic effect of alcohol) because our study was focused on poisoning by medications.

Various demographic characteristics were collected, such as age, sex, admission date, type of poisoning (deliberate self-poisoning, accidental poisoning, or not reported), and outcome (discharge or death). Gender is based on self-report of male or female. The age at admission was rounded to the nearest year. Hospital admissions were classified according to seasons as Spring = March 21 to June 20; Summer = June 21 to September 22; Autumn = September 23 to December 21; Winter = December 22 to March 20.

Using a case chart, we assigned the ATC codes to each medication and categorized the drugs into nine categories. Two further categories were established for alcohol and illicit substances. Multi-substance ingestion cases were assigned codes associated with every substance consumed. The causality assessment for poisoning was enhanced by excluding cases whose chart reviews included medications other than the ATC code N.

This study assessed prevalence, age and gender distribution of acute poisoning by agents affecting the nervous system. Using chi-square independent testing, we compared categorical variables across genders and age groups. Additionally, we determined statistical significance (*p*-value) between the median ages of the male and female groups using the Mann-Whitney U test. We examined trends in the number of patients poisoned by these agents and gender differences over a period of five years. A z-test was used to test the relative significance of the overall trends, while a Mann-Kendall trend test was used to assess their significance. The levels of *p* < 0.05 were accepted as statistically significant. Data analysis were performed using Excel 2017 and R version 4.3.0 [28].

## Results

From 49,505 patients admitted to the hospital for acute poisoning during the study period who took at least one substance acting on the nervous system, 29,083 met our inclusion criteria. Patients were predominantly male (*n* = 16,657; 57.27%), with a male-to-female ratio of 1.3:1. Patient ages ranged from 1 month to 97 years with a median age of 28 years (29 in men and 26 in women, *p* < 0.0001). Of the 29,083 patients, 77% were aged less than 40 years (72.47% of men and 83.10% of women, *p* < 0.001).

The majority of patients (*n* = 21,374, 73.49%) were admitted for self-poisoning, compared to 1017 (3.50%) cases for accidental poisoning. Intention of poisoning was not recorded in 6692 cases (23.01%). 11,497 patients (39.53%) had a previous history of poisoning.

We identified 85 medications which we classified into 14 groups based on the second or third level of their respective ATC code. Further, we used two groups of illicit drugs and alcohol for the nine other agents (Supplementary file). The overall trend of acute poisoning with drug used in addictive disorders (*p* = 0.008), opioids (*p* = 0.008), and alcohol (*p* = 0.02) increased during the study period, whereas it decreased with benzodiazepines (*p* = 0.03) and antidepressants (*p* = 0.008). During the study period, no significant change was observed in admissions of patients with acute poisoning caused by all different medications and substances (*p* = 0.26), illicit drugs (*p* = 0.45), antiepileptics (*p* = 0.45), antipsychotics (*p* = 0.06), analgesics (*p* = 0.08), hypnotics and sedatives (*p* = 0.06), psychostimulants (*p* = 0.08), antiparkinson (*p* = 0.45) and anxiolytics (*p* = 0.85) (Figs. 2 and 3).

Regardless of age or gender, methadone (in the category of drug used in addictive disorders) was the most frequently used poisoning medication with 5292 hospitalized cases, followed by clonazepam in the antiepileptic class (2488 cases) and alprazolam in the benzodiazepine class (2406). Among illicit drugs, opium (2690) was the most commonly used substance, followed by methamphetamine (1979) and crack cocaine (736).

Deliberate poisoning was most commonly caused by methadone (2820), alprazolam (2104), and clonazepam (2082). Over half of the accidental poisonings were caused by opium use.

We found that 395 (1.36%) of the studied cases had expired. More than half of the deaths (219/395; 55.44%) were caused by illicit drugs and 33.42% by opium (132/395). Drugs used in addictive disorders were the most common cause of death among medications. Acute poisoning by anxiolytics, other nervous system drugs, anesthetics and antidementia have not been associated with death (Table 1).

**Table 1:** Gender-based comparison of acute poisoning and relevant death by class of agent affecting the nervous system

Medication/drug Class	No. of admitted patients (%)				Died due to poisoning			
	Female	Male	Total	*p* value	Female	Male	Total	*p* value^a^
Illicit drugs	1103 (18.14)	4979 (81.86)	6082 (20.91)	< 0.0001	25 (11.42)	194 (88.58)	219 (3.60)	< 0.0001
Benzodiazepine	3081 (58.99)	2142 (41.01)	5223 (17.96)	< 0.0001	9 (33.33)	18 (66.67)	27 (0.52)	0.322
Drugs Used in Addictive Disorders	1296 (24.49)	3996 (75.51)	5292 (18.20)	< 0.0001	10 (12.50)	70 (87.50)	80 (1.55)	< 0.0001
Antiepileptics	2639 (58.62)	1863 (41.38)	4502 (15.48)	< 0.0001	11 (47.83)	12 (52.17)	23 (0.51)	0.624
Opioids	1105 (38.85)	1739 (61.15)	2844 (9.78)	< 0.0001	3 (20.00)	12 (80.00)	15 (0.50)	0.075
Antidepressants	1926 (71.89)	753 (28.11)	2679 (9.21)	< 0.0001	7 (77.78)	2 (22.22)	9 (0.34)	0.033
Antipsychotics	656 (56.12)	513 (43.88)	1169 (4.02)	< 0.0001	3 (30.00)	7 (70.00)	10 (0.86)	0.417
Analgesics	268 (64.89)	145 (35.11)	413 (1.42)	< 0.0001	1 (100.00)	0 (0.00)	1 (0.24)	0.246
Hypnotics and Sedatives	161 (58.33)	115 (41.67)	276 (0.95)	< 0.0001	0 (0.00)	1 (100.00)	1 (0.36)	0.389
Alcohol	26 (12.32)	185 (87.68)	211 (0.73)	< 0.0001	0 (0.00)	3 (100.00)	3 (1.42)	0.133
Psychostimulants	68 (32.69)	139 (67.15)	207 (0.71)	0.003	0 (0.00)	5 (100.00)	5 (2.40)	0.053
Antiparkinson	56 (44.44)	70 (55.56)	126 (0.43)	0.696	1 (50.00)	1 (50.00)	2 (1.59)	0.833
Anxiolytics	35 (71.43)	14 (28.57)	49 (0.17)	< 0.0001	0 (0.00)	0 (0.00)	0 (0.00)	-
Other Nervous System Drugs	3 (60.00)	2 (40.00)	5 (0.02)	0.435	0 (0.00)	0 (0.00)	0 (0.00)	-
Anesthetics	1 (33.33)	2 (66.67)	3 (0.01)	0.741	0 (0.00)	0 (0.00)	0 (0.00)	-
Antidementia	2 (100.00)	0 (0.00)	2 (0.01)	0.101	0 (0.00)	0 (0.00)	0 (0.00)	-
**Total**	**12,426 (42.73)**	**16,657 (57.27)**	**29,083 (100.00)**	**-**	**70 (17.72)**	**325 (82.28)**	**395 (1.36)**	**-**

^a^According to the results of a X^2^-test

### Acute poisoning and gender

A chi-square test of independence revealed that gender was significantly associated with the intention of poisoning, repeated exposure, discharge of patients, and hospitalization year, but not with the season of hospitalization (Table 2).

**Table 2:** Gender-based comparison of patient characteristics

Description	Total *N* = 29,083	Female (%) *n* = 12,426	Male (%) *n* = 16,657	*p*-value^a^
**Circumstance of exposure**				< 0.0001
Deliberate self-poisoning	21,374	9817 (45.93)	11,557 (54.07)	< 0.0001
Accidental	1017	342 (33.63)	675 (66.37)	< 0.0001
Not reported	6692	2267 (33.88)	4425 (66.12)	< 0.0001
**Number of hospital admission**				< 0.0001
Once	17,586	7290 (41.45)	10,296 (58.55)	< 0.0001
More than once	11,497	5136 (44.67)	6361 (55.33)	< 0.0001
**Patients discharge**				< 0.0001
Following treatment	28,688	12,356 (43.07)	16,332 (56.93)	< 0.0001
Due to death	395	70 (17.72)	325 (82.28)	< 0.0001
**Year of hospital admission**				0.0
2010	4768	2171 (45.53)	2597 (54.47)	< 0.0001
2011	4719	2027 (42.95)	2692 (57.05)	0.726
2012	4542	2100 (46.24(	2442 (53.76)	< 0.0001
2013	4808	2094 (43.55)	2714 (56.45)	0.204
2014	5126	2106 (41.08)	3020 (58.92)	< 0.05
2015	5120	1928 (37.66)	3192 (62.34)	< 0.0001
**Season of hospital admission**				0.143
Spring	7574	3308 (43.68)	4266 (56.32)	0.052
Summer	7891	3306 (41.90)	4585 (58.10)	0.080
Autumn	6820	2929 (42.95)	3891 (57.05)	0.674
Winter	6798	2883 (42.41)	3915 (57.59)	0.548

^a^According to the results of a X^2^-test

It was found that most women (79.00%) and men (69.38%) were admitted following an intentional poisoning (*p* < 0.001), and 45.10% of women and 43.26% of men had a history of admission following poisoning (*p* = 0.007). Accidental poisoning was more common in men (675/1017; 66.37%) than in women (342/1017; 33.63%) (*p* < 0.00001). The overall trend of deliberate self-poisoning decreased in women over this six years period (*p* = 0.02), while in men, it did not change (*p* = 0.45).

In deliberate poisonings, alprazolam (1397), clonazepam (1074) and diazepam (578) were most commonly used by women, while methadone (2255), tramadol (1149) and opium (1146) were most commonly used by men.

**Fig. 1:**
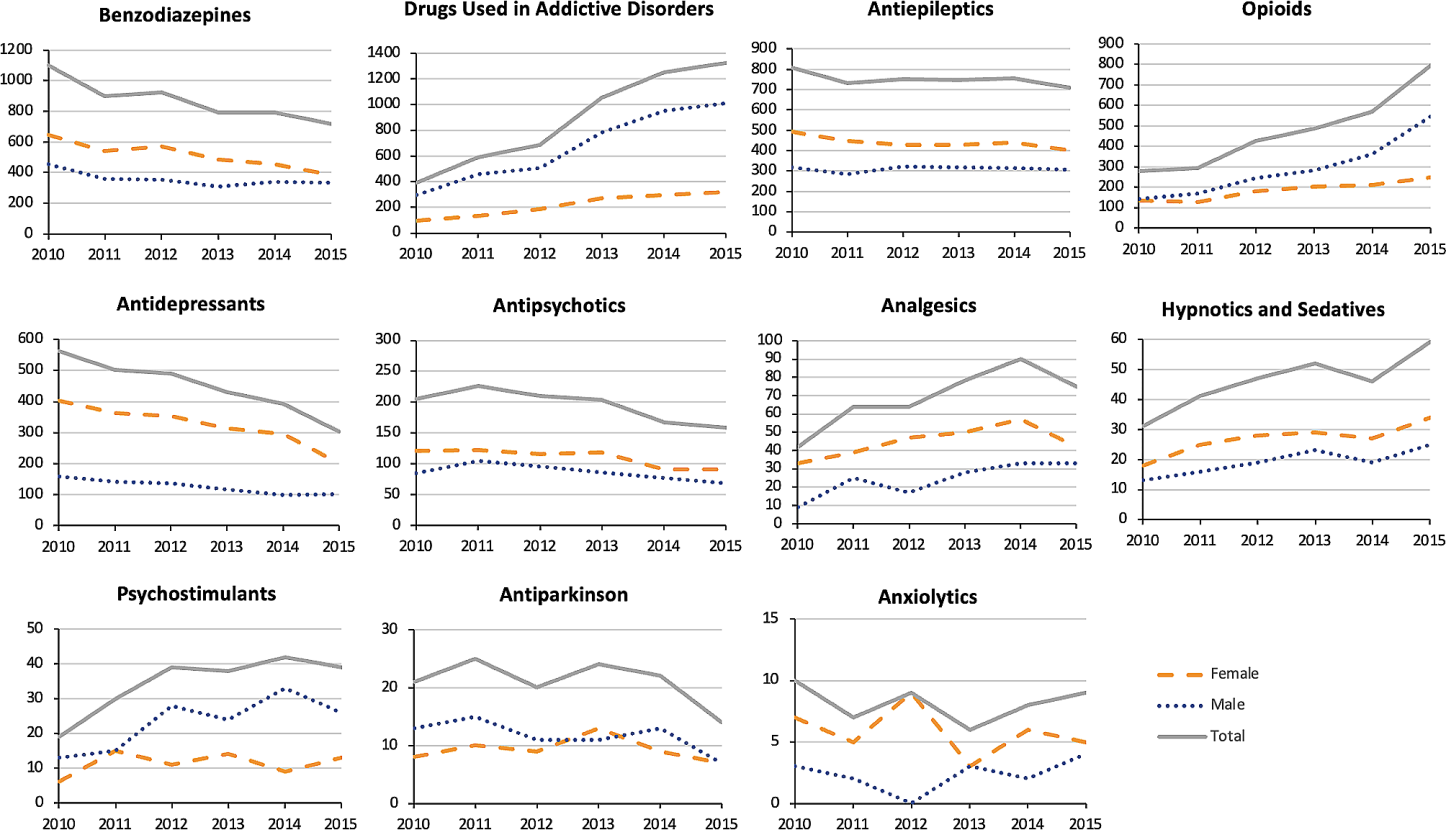
Trend in poisoning caused by medications that affect the nervous system, 2010–2015, gender-specific analysis sorted by the total number of admitted patients

**Fig. 2:**
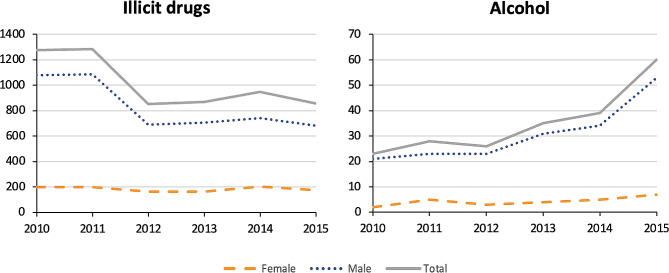
Trend in poisoning by illicit drugs and alcohol, 2010–2015, gender-specific analysis

A significant gender difference was found in all classes of medications and substances except antiparkinson, anesthetics, antidementia and other nervous system drugs (Table 1). The trend of gender differences for acute poisoning with drugs used in addictive disorders (*p* = 0.02), opioids (*p* = 0.008), and alcohol (*p* = 0.02) was increasing, but declined for antidepressants (*p* = 0.008) and did not change across other classes (Figs. 1 and 2).

A significant proportion of men died from acute poisoning caused by nervous system agents (325 men (1.95%) compared to 70 women (0.56%); *p* < 0.001). Among the 288 deaths caused by deliberate poisoning, 191 were men and 37 were women (male-to-female ratio of 4.3:1). Accidental poisoning resulted in 20 deaths (2.07% in men and 1.75% in women). Death due to poisoning with illicit drugs and drugs used in addictive disorders was significantly higher in men (*p* < 0.0001) as it was higher in women following acute poisoning with antidepressants (*p* = 0.03) (Table 1).

More deaths were caused by methamphetamine, methadone, and opium poisoning in men than any other agent, likely as a result of their frequent usage among men. The most common causes of death among women were methadone, opium, and clonazepam, while the main cause of acute poisoning was alprazolam, clonazepam, and methadone. Methadone and opium deaths were significantly different in women and men (10/1267; 0.79% vs. 69/3852; 1.79% *p* = 0.01 and 19/605; 3.14% vs. 113/2085; 5.42% *p* = 0.02, respectively).

### Acute poisoning, gender and age

**Fig. 3:**
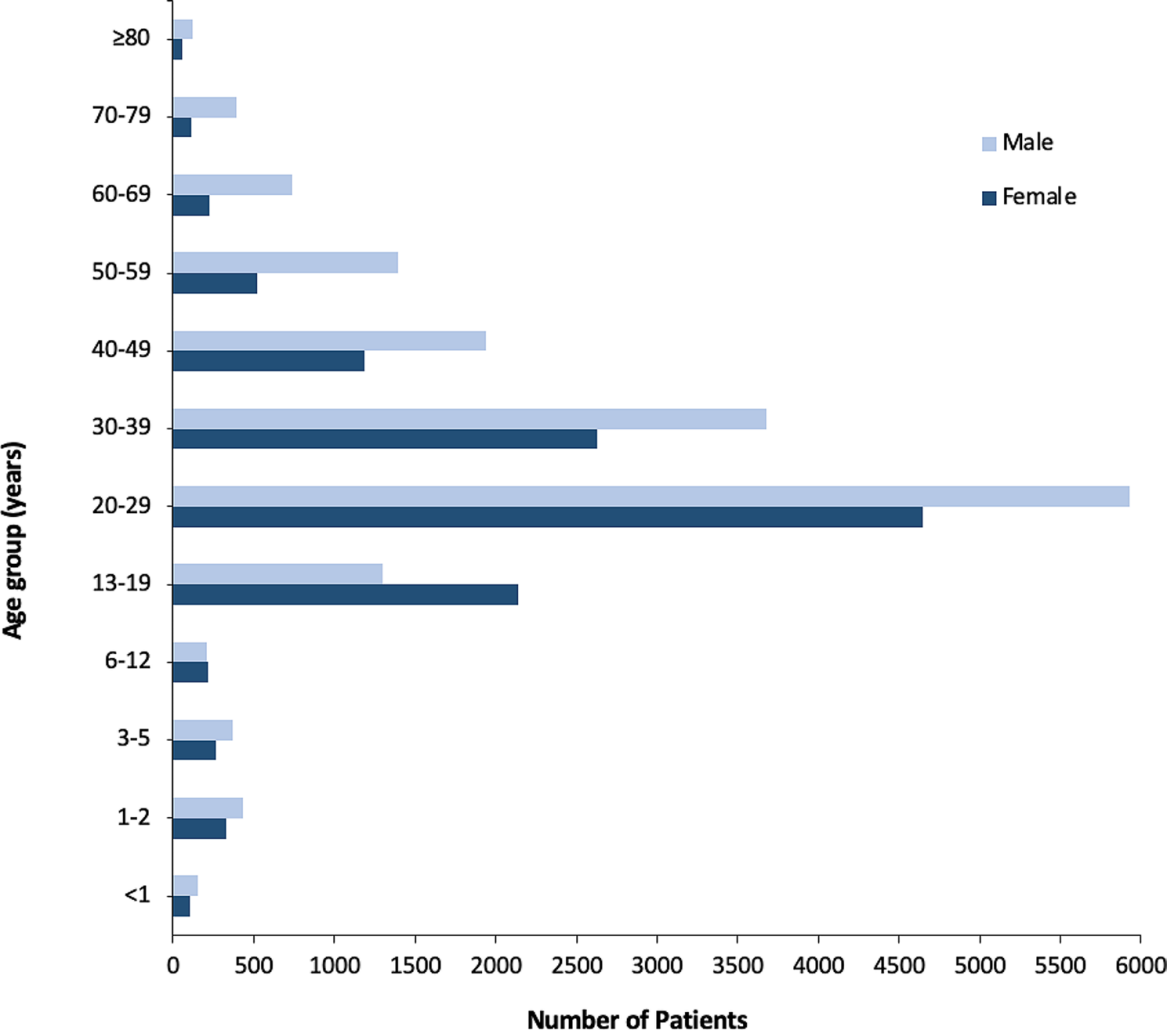
Distribution of poisoning patients at Loghman-Hakim Hospital by age and gender, 2010–2015

Overall, there were significant gender differences in age (females age range was 1 month to 97 years with a mean equal to 26 years, while males age range was 1 month to 94 years with a mean equal to 29 years; *p* < 0.0001). The age and gender distribution are shown in Fig. 3.

Patients aged 20 to 29 accounted for the highest percentage of acute poisonings (*n* = 10,576, 36.36%) compared to other age groups. Poisoning was more prevalent among men than women in all age groups except those 6 to 19 years old (2356/3858; 61.07% vs. 1502/3858; 38.93%, *p* < 0.00001).

Out of 11,557 men who attempted suicide, 4661 (40.33%) were between the ages of 20 and 29. In both men and women, deliberate self-poisoning and accidental poisoning were more prevalent in those aged 20–29 years old. Only 8.95% of accidental poisonings occurred in children under the age of 13.

**Fig. 4:**
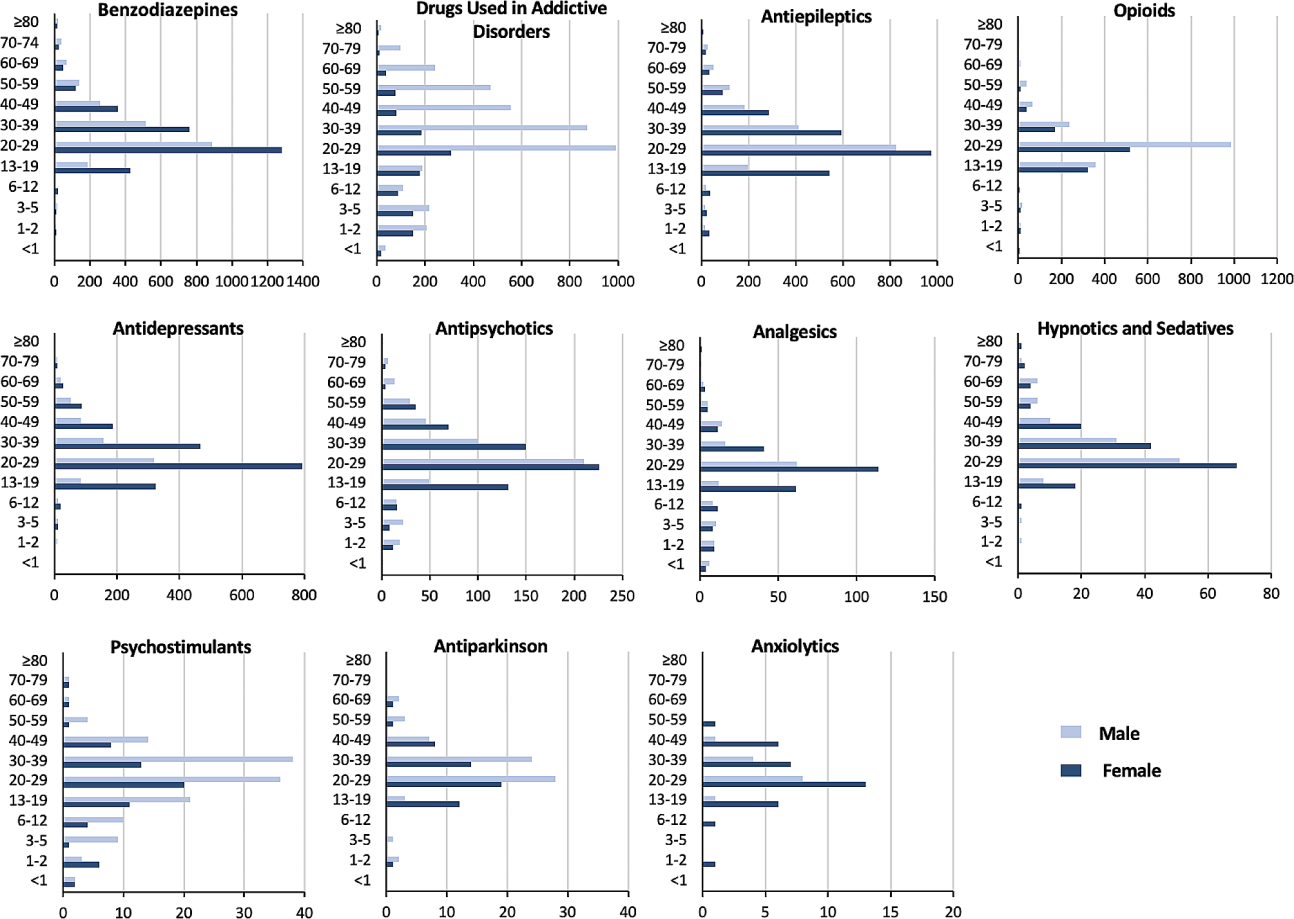
Distribution of poisonings caused by medications that affect the nervous system by age and gender

**Fig. 5:**
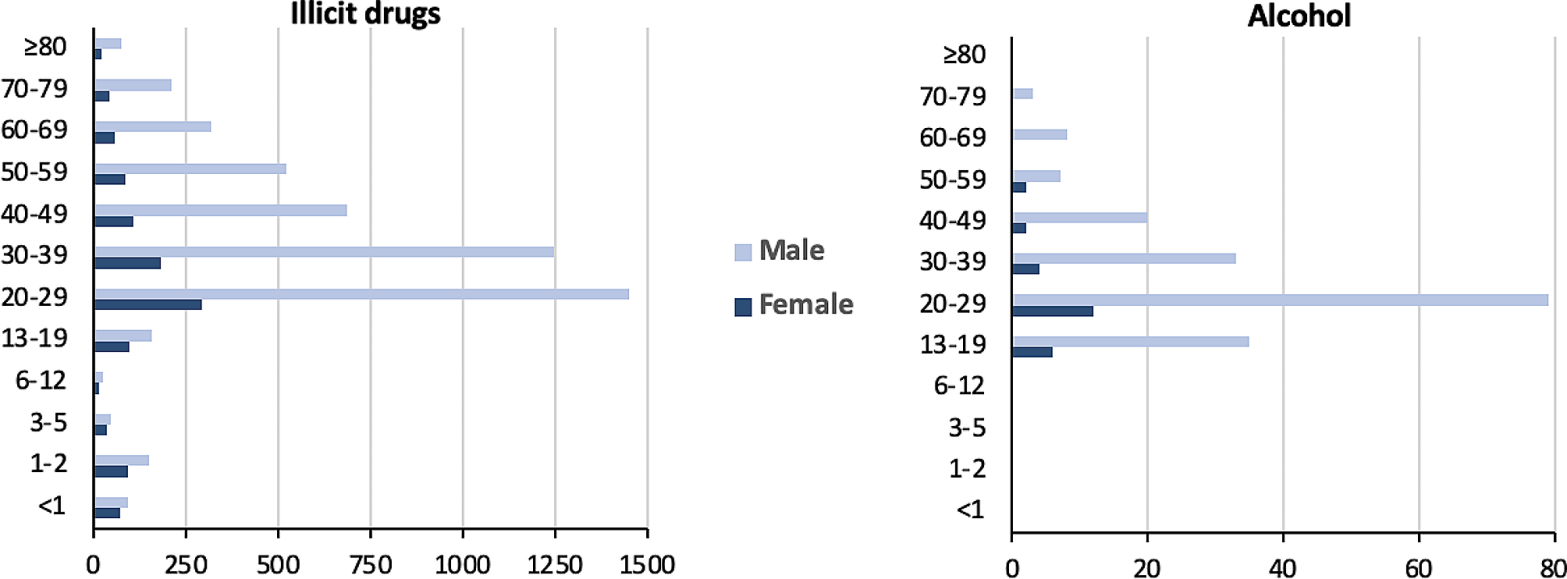
Distribution of poisoning by illicit drugs and alcohol by age and gender

In women aged 20–59, benzodiazepines were most frequently responsible for acute poisoning while illicit drugs were the most common cause among women aged 60 and over. In men over 20 years of age, illicit drugs were more likely to cause acute poisoning. As a result of poisoning by illicit drugs, men were admitted to hospitals 4.5 times more often than women, and 54% of those men were between the ages of 20 and 39. In infants under 12 months of age, illicit drugs were the most common cause of acute poisoning in both genders. The most prevalent agents in children aged 1 to 12 years were drugs used in addictive disorders in both females and males. Nevertheless, girls and boys aged 13–19 years were most likely to be acutely poisoned by antiepileptics and opioids, respectively. According to the Mann-Withney U test, there was a significant age difference between females and males with regard to acute poisoning with illicit drugs (median age in females: 27 years vs. 33 years in males; *p* < 0.0001), benzodiazepines (median age in females: 28 years vs. 29 years in males; *p* < 0.0001), drugs used in addictive disorders (median age in females: 21 years vs. 32 years in males; *p* < 0.0001), antiepileptics (median age in females: 26 years vs. 27 years in males; *p* < 0.001), opioids (median age in both genders: 23 years; *p* < 0.01) and antidepressants (median age in both genders: 27 years; *p* = 0.02) (Figs. 4 and 5).

In both genders, children aged 12 years and younger were primarily poisoned with methadone, opium, and methamphetamine. Girls and boys aged 13–19 years were poisoned most often by alprazolam and tramadol, respectively, with significant gender differences (*p* < 0.001). The most prevalent poisonings in adults aged 20–49 were alprazolam and clonazepam in women and methadone and methamphetamine in men, with significant gender differences (*p* < 0.001). Opium, methadone, clonazepam and alprazolam were the most frequent agents in patients 50 years of age and older, with significant gender differences (*p* < 0.001).

Men aged 30–39 accounted for 17.72% of deaths caused by acute poisoning by nervous system agents (70/395), mainly by methamphetamine, opium, and methadone. Death was also common among men aged 70 and older, with 64.61% resulting from opium poisoning. Female deaths were more common in 20–29 years with alprazolam and 70 years and older with opium poisoning.

## Discussion

We studied gender differences in incidence of acute poisoning with drugs and medications affecting nervous system per age category of patients admitted to the largest referral center of toxicology in Tehran, in pursuit of a study reported these agents were accounted for 36.68% (39,708/108,265) poisoning from 2006 to 2011 in LHHPC [29]. The gender differences in acute poisoning cases have been examined in various studies conducted in Iran. Inconsistencies in the findings suggest the need for further research to better understand the causes of these gender disparities. A noteworthy aspect of our study is that we had a specific target group of poisoning patients with a large sample size over a six-year period.

In line with previous studies, this study found that men are more likely than women to be acutely poisoned and to suffer deliberate and accidental poisoning [6, 27, 29–34]. However, two previous studies in LHHPC [35, 36] and others in other cities of Iran reported higher female to male ratios in acute poisoned patients [2, 26, 36–39].

In the present study, statistically significant differences were found between genders with respect to age. Men had a higher median age of 29 years than females at 26 years. Additionally, individuals aged 20 to 29 years were more likely to suffer acute poisoning. The prevalence of poisoning was higher among men in various age groups, except for the 6 to 19 years age group, which was contrary to other studies with smaller sample sizes [33–35].

Most women (79.00%) and men (69.38%) were admitted after intentionally poisoning, with 45.10% of women and 43.26% of men having history of admission due to poisoning. Furthermore, a significant proportion of male suicide attempts (40.33%) occurred between 20 and 29 years old. A study conducted at LHHPC in 2003 found that suicide poisoning represented 79% of all poisoning cases in men and women [27]. An analysis of 71,589 poisoned patients in Mashhad concluded that 54.4% were intentional and most prevalent among those aged 18–29 [37]. In a study conducted in 1208 poisoned people in Urmia in 2013–2016, the most cases of poisoning (86%) were intentional [40]. There were 8.95% of accidental poisoning incidents involving children under 13 years old, contrary to the studies in other countries [41, 42].

Gender-specific variations were seen in the trend of intentional poisoning, particularly suicide attempts. Among women, the overall trend of intentional poisoning decreased, which is consistent with the WHO report on age-standardized suicide rates (from 3.76 per 100,000 population in 2010 to 3.11 per 100,000 population in 2015). Our study, however, found inconsistent results from the WHO report regarding intentional poisoning in men. It is possible that these variations are influenced by the specific agents that were assessed in our study, and further investigation is warranted [43].

There was no significant seasonal difference in the rate of intentional or unintentional poisonings in this study. However, this finding differs from a previous study by Ghafarian Shirazi, et al. reporting a higher suicide rate during the summer (35.2%), about 13% higher than in other seasons [44].

Our observed mortality rate was 1.36% (398 out of 29,465). It confirms the 2003 study, which found that 318 out of 10,206 (1.3%) patients with acute poisoning, regardless of the type of poisoning, died in this hospital [27].

It was found that death was significantly more common in men (82.28%) and that men aged 30–39 accounted for 17.72% of all nervous system agent deaths, primarily methamphetamine, opium, and methadone. According to previous research [34], men are more likely to die from acute poisoning regardless of their intention. Among 10,206 patients referred with acute poisoning to LHHPC in 2003, opioids (40.6% of deaths) were the leading causes of death [27].

According to our findings, methadone was the most common drug that caused hospitalization (5119 poisoned patients). Methadone has been identified as the most common agent of acute poisoning in other studies as well [29, 30, 34]. Our previous study showed that methadone poisoning increased from 2000 to 2010 [45]. The systemic review of methadone-related poisoning in Iran found that it caused 10.4% of adult cases and 16.0% of child cases [46].

Patients between the ages of 20 and 39 were more likely to be poisoned by agents used in addictive disorders, including methadone. It is consistent with a study conducted in Isfahan from 2010 to 2012, which found that 60% of patients poisoned with methadone were between 20 and 39 [47]. A 7.7-fold increase in methadone-associated deaths in Iran has been observed between 2009 and 2015 (*p* < 0.05) [48]. Methadone poisoning rates may be high in Iran as a result of unstandardized methadone maintenance treatment programs and its availability in the black markets [49]. Accordingly, the probability of accidental poisoning might be increased by family members of methadone-dependent patients.

To our knowledge, this is the first study conducted in Iran to examine whether acute poisoning by the nervous system agents differs by gender and age. This study, however, had limitations: the retrospective nature of the study, incomplete records, lack of random sampling, and lack of justification for medication utilization limited the generalizability of the findings. In addition, the records of patients hospitalized due to alcohol-related toxic effects were not included in our study, which may have resulted in an underestimate of the true incidence of acute alcohol poisoning.

## Conclusion

In summary, methadone was the most common cause of acute poisoning by nervous system agents, followed by illicit substances. Even though most poisoning cases were intentional suicides, the mortality rate of the study population was low. Poisoning was more prevalent among young people, especially men. To reduce poisonings, measures such as gender and age-specific educational programs, and the availability of these drugs, can be implemented.

### Electronic supplementary material

Below is the link to the electronic supplementary material.

Supplementary Material 1

## Data Availability

The data that support the findings of this study are available from the corresponding author, BM, upon reasonable request.

## Abbreviations

LHHPC: Loghman-Hakim Hospital Poison Center
ATC code: Anatomical Therapeutic Chemical code
ICD-10: International Classification of Diseases, Tenth Revision

## References

[1] Molla YM, Belachew KD, Ayehu GW, Teshome AA (2022). Acute poisoning in children in Ethiopia: a cross-sectional study. Sci Rep.

[2] Naseri K, Kiani Z, Sajadi ZS, Mehrpour O, Javadmoosavi SY, Forouzanfar F (2023). Pharmaceutical toxicity is a common pattern of inpatient acute poisonings in Birjand City, East of Iran. Sci Rep.

[3] Sharma R, Neelanjana, Rawat N, Panwar N (2019). Mortality and morbidity associated with acute poisoning cases in north-east India: a retrospective study. J Family Med Prim Care.

[4] Zhang Y, Yu B, Wang N, Li T (2018). Acute poisoning in Shenyang, China: a retrospective and descriptive study from 2012 to 2016. BMJ Open.

[5] Abd-Elhaleem ZAE, Al Muqhem B (2014). Pattern of acute poisoning in Al Majmaah region, Saudi Arabia. Am J Clin Exp Med.

[6] Alinejad S, Zamani N, Abdollahi M, Mehrpour O (2017). A narrative review of Acute Adult Poisoning in Iran. Iran J Med Sci.

[7] Chelkeba L, Mulatu A, Feyissa D, Bekele F, Tesfaye BT (2018). Patterns and epidemiology of acute poisoning in Ethiopia: systematic review of observational studies. Arch Public Health.

[8] Gummin DD, Mowry JB, Beuhler MC, Spyker DA, Bronstein AC, Rivers LJ et al. 2020 annual report of the american association of poison control centers’ National Poison Data System (NPDS): 38th Annual Report. Clin Toxicol (Phila). 2021;59(12):1282–1501. 10.1080/15563650.2021.1989785. 10.1080/15563650.2021.1989785 34890263

[9] Al-Jelaify M, AlHomidah S (2021). The Individualized Management Approach for Acute Poisoning. Adv Pharmacol Sci.

[10] Eizadi-Mood N, Mahvari R, Akafzadeh Savari M, Mohammadbeigi E, Feizi A, Mirmoghtadaei P (2023). Acute pesticide poisoning in the central part of Iran: a 4-year cross-sectional study. SAGE Open Med.

[11] Rönkä S, Katainen A (2017). Non-medical use of prescription drugs among illicit drug users: a case study on an online drug forum. Int J Drug Policy.

[12] Murtazina RZ, Kuvarzin SR, Gainetdinov RR (2021). TAARs and neurodegenerative and Psychiatric disorders.

[13] Ruha AM, Levine M (2014). Central nervous system toxicity. Emerg Med Clin N Am.

[14] Jones CM, Mack KA, Paulozzi LJ (2013). Pharmaceutical overdose deaths, United States, 2010. JAMA.

[15] Mowry JB, Spyker DA, Brooks DE et al. 2014 Annual report of the American Association of Poison Control Centers’ National Poison Data System (NPDS): 32nd annual report. Clin Toxicol. 2015;53(10):962–1147. 10.3109/15563650.2015.1102927. 10.3109/15563650.2015.1102927 26624241

[16] Kaka RA, Ghanem MAA, Sigairon MEE, Elabedin HZ, Mostafa H (2022). A retrospective analysis of Acute Poisoning cases admitted to Alexandria poison Center: Pattern and Outcome. Asia Pac J Med Toxicol.

[17] Resiere D, Kallel H, Oxybel O, Chabartier C, Florentin J, Brouste Y (2020). Clinical and epidemiological characteristics of severe acute adult poisoning cases in Martinique: implicated toxic exposures and their outcomes. Toxics.

[18] Bruno T, Pharr JR (2017). Retrospective case series analysis of characteristics and trends in unintentional pharmaceutical drug poisoning by methadone, opioid analgesics, antidepressants and benzodiazepines in Clark County, NV 2009-13. J Public Health.

[19] Mauvais-Jarvis F, Berthold HK, Campesi I, Carrero JJ, Dakal S, Franconi F (2021). Sex- and gender-based pharmacological response to drugs. Pharmacol Rev.

[20] Miró Ò, Waring WS, Dargan PI, Wood DM, Dines AM, Yates C (2021). Variation of drugs involved in acute drug toxicity presentations based on age and sex: an epidemiological approach based on European emergency departments. Clin Toxicol.

[21] Nicolson TJ, Mellor HR, Roberts RR (2010). Gender differences in drug toxicity. Trends Pharmacol Sci.

[22] Kokras N, Dalla C, Papadopoulou-Daifoti Z (2011). Sex differences in pharmacokinetics of antidepressants. Expert Opin Drug Metab Toxicol.

[23] Wightman RS, Perrone J, Scagos R, Hallowell BD, Krieger M, Li Y (2021). Toxicological and pharmacologic sex differences in unintentional or undetermined opioid overdose death. Drug Alcohol Depend.

[24] Alnasser SM (2022). Drug and chemical poisoning patterns in Makkah Region, Saudi Arabia. Drug Res.

[25] Bresin K, Schoenleber M, Alnasser SM (2015). Gender differences in the prevalence of nonsuicidal self-injury: a meta-analysis. Clin Psychol Rev.

[26] Najjari F, Ramazannejad P, Ahmadi A, Amini Z (2016). Epidemiological study of poisoning in patients referring educational and clinical center of Ayatollah Kashani hospital, Shahrekord (West of Iran) throughout 2008–2014. Int J Med Toxicol Forensic Med.

[27] Shadnia S, Esmaily H, Sasanian G, Pajoumand A, Hassanian-Moghaddam H, Abdollahi M (2007). Pattern of acute poisoning in Tehran-Iran in 2003. Hum Exp Toxicol.

[28] R Core Team. A language and environment for statistical computing. 2022. R Foundation for Statistical Computing, Vienna, Austria. https://www.R-project.org/.

[29] Hassanian-Moghaddam H, Zamani N, Rahimi M, Shadnia S, Pajoumand A, Sarjami S (2014). Acute adult and adolescent poisoning in Tehran, Iran; the epidemiologic trend between 2006 and 2011. Arch Iran Med.

[30] Afzali S, Moradi A, Alinaghizadeh H (2020). Epidemiologic characteristics and outcomes of drugs poisoning in the Hamadan, Iran:(2015–2019). Asia Pac J Med Toxicol.

[31] Banaye Yazdipour A, Moshiri M, Dadpour B, Sarbaz M, Heydarian Miri H, Hajebi Khaniki S (2022). The trend of top five types of poisonings in hospitalized patients based on ICD-10 in the northeast of Iran during 2012–2018: a cross-sectional study. Health Sci Rep.

[32] Hadeiy SK, Parhizgar P, Hassanian-Moghaddam H, Zamani N, Khoshkar A, Kolahi AA (2022). Trends of acute drug and chemical toxicities in adults and adolescents in Tehran, Iran between 2012 and 2018: a retrospective chart review. Drug Chem Toxicol.

[33] Mehrpour O, Akbari A, Jahani F, Amirabadizadeh A, Allahyari E, Mansouri B (2018). Epidemiological and clinical profiles of acute poisoning in patients admitted to the intensive care unit in eastern Iran (2010 to 2017). BMC Emerg Med.

[34] Torkashvand F, Sheikh Fathollahi M, Shamsi S, Kamali M, Rezaeian M (2015). Evaluating the pattern of acute poisoning in cases referred to the Emergency Department of Ali-Ebn Abi Taleb Hospital of Rafsanjan from October 2013 to September 2014. J Rafsanjan Univ Med Sci.

[35] Abdollahi M, Jalali N, Sabzevari O, Hoseini R, Ghanea T (1997). A retrospective study of poisoning in Tehran. J Toxicol Clin Toxicol.

[36] Ghazinour M, Emami H, Richter J, Abdollahi M, Pazhumand A (2009). Age and gender differences in the use of various poisoning methods for deliberate parasuicide cases admitted to Loghman hospital in Tehran (2000–2004). Suicide Life Threat Behav.

[37] Afshari R, Majdzadeh R, Balali-Mood M (2004). Pattern of acute poisonings in Mashhad, Iran 1993–2000. J Toxicol Clin Toxicol.

[38] Islambulchilar M, Islambulchilar Z, Kargar-Maher MH (2009). Acute adult poisoning cases admitted to a university hospital in Tabriz, Iran. Hum Exp Toxicol.

[39] Moradi-asl E, Nikookar H, Danandehpor P, Vakili F, Asadyian M, Adham D (2020). Study of mortality rate and poisoning status by using pesticides, drugs, and chemicals in Meshkinshahr, Ardabil province. Iran J Health Sci.

[40] Zare Fazlohahi ZM, Shaikhi M (2010). Epidemiology of adult poisoning in Talegani hospital of Urmia 1383–1386. Nurs Midwifery J.

[41] Koh SH, Tan KHB, Ganapathy S (2018). Epidemiology of paediatric poisoning presenting to a children’s emergency department in Singapore over a five-year period. Singap Med J.

[42] Thanacoody R, Anderson M (2020). Epidemiology of poisoning. Medicine.

[43] World Health Organization. Age-standardised Suicide Rates for 2000–2019. 2021. https://www.who.int/data/gho/data/indicators/indicator-details/GHO/age-standardized-suicide-rates-(per-100-000-population).

[44] Ghafarian Shirazi HR, Hosseini M, Zoladl M, Malekzadeh M, Momeninejad M, Noorian K (2012). Suicide in the Islamic Republic of Iran: an integrated analysis from 1981 to 2007. East Mediterr Health J.

[45] Soltaninejad K, Hassanian-Moghaddam H, Shadnia S (2014). Methadone related poisoning on the rise in Tehran, Iran. Asia Pac J Med Toxicol.

[46] Rostam-Abadi Y, Gholami J, Noroozi A, Ansari M, Baheshmat S, Hamzehzadeh M (2022). Public health risks associated with methadone in Iran: a systematic review and meta-analysis. Int J Drug Policy.

[47] Taheri F, Yaraghi A, Sabzghabaee AM, Moudi M, Eizadi-Mood N, Gheshlaghi F (2013). Methadone toxicity in a poisoning referral center. J Res Pharm Pract.

[48] Akhgari M, Amini-Shirazi N, Iravani FS (2018). Forensic toxicology perspectives of methadone-associated deaths in Tehran, Iran, a 7-year overview. Basic Clin Pharmacol Toxicol.

[49] Shadnia S, Rahimi M, Hassanian-Moghaddam H, Soltaninejad K, Noroozi A. Methadone toxicity: comparing tablet and syrup formulations during a decade in an academic poison center of Iran. Clin Toxicol. 2013 Sep–Oct;51(8):777–82. 10.3109/15563650.2013.830732 10.3109/15563650.2013.830732 23972442

