# Supplementary material for: Age- and gender-specific acute poisoning with drugs and medications affecting nervous system
Source: BMC Pharmacol Toxicol. 2024 Jul 1;25:37. doi: 10.1186/s40360-024-00759-1 (PMC11218142; doi:10.1186/s40360-024-00759-1)
Supplement: Supplementary file 1 — Supplementary Material 1 [file 40360_2024_759_MOESM1_ESM.docx]

**Age-and gender-specific acute poisoning with drugs and medications affecting nervous system**

**Supplementary**

| Category | Medication | ATC code |
| --- | --- | --- |
| Drugs used in Addictive Disorders | Methadone | N07BC02 |
| Drugs used in Addictive Disorders | Naltrexone | N07BB04 |
| Drugs used in Addictive Disorders | Buprenorphine | N07BC01 |
| Alcohol | Alcohol | - |
| Analgesics | Acetylsalicylic acid | N02BA01 |
| Analgesics | Codeine Phosphate | N02AJ06 |
| Analgesics | Dihydroergotamine | N02CA01 |
| Analgesics | Ergotamine + Caffeine | N02CA52 |
| Analgesics | Paracetamol | N02BE01 |
| Analgesics | Paracetamol+Caffeine | N02BE |
| Analgesics | Paracetamol+Caffeine+Aspirin | - |
| Analgesics | Paracetamol+Caffeine+Ibuprofen | - |
| Analgesics | Sumatriptan | N02CC01 |
| Anesthetics | Bupivacaine | N01BB01 |
| Anesthetics | Ketamine | N01AX03 |
| Antidementia | Donepezil | N06DA02 |
| Antidepressants | Amitriptyline | N06AA09 |
| Antidepressants | Bupropion | N06AX12 |
| Antidepressants | Citalopram | N06AB04 |
| Antidepressants | Clomipramine | N06AA04 |
| Antidepressants | Desipramine | N06AA01 |
| Antidepressants | Duloxetine | N06AX21 |
| Antidepressants | Fluoxetine | N06AB03 |
| Antidepressants | Fluvoxamine | N06AB08 |
| Antidepressants | Imipramine | N06AA02 |
| Antidepressants | Isocarboxazid | N06AF01 |
| Antidepressants | Maprotiline | N06AA21 |
| Antidepressants | Nortriptyline | N06AA10 |
| Antidepressants | Sertraline | N06AB06 |
| Antidepressants | Tranylcypromine | N06AF04 |
| Antidepressants | Trazodone | N06AX05 |
| Antidepressants | Trimipramine | N06AA06 |
| Antidepressants | Venlafaxine | N06AX16 |
| Antiepileptics | Carbamazepine | N03AF01 |
| Antiepileptics | Clonazepam | N03AE01 |
| Antiepileptics | Gabapentin | N02BF01 |
| Antiepileptics | Lamotrigine | N03AX09 |
| Antiepileptics | Phenobarbital | N03AA02 |
| Antiepileptics | Phenytoin | N03AB02 |
| Antiepileptics | Pregabalin | N02BF02 |
| Antiepileptics | Primidone | N03AA03 |
| Antiepileptics | Topiramate | N03AX11 |
| Antiepileptics | Valproic Acid | N03AG01 |
| Antiparkinson | Amantadine | N04BB01 |
| Antiparkinson | Biperiden | N04AA02 |
| Antiparkinson | Levodopa | N04BA01 |
| Antiparkinson | Pramipexole | N04BC05 |
| Antiparkinson | Trihexyphenidyl | N04AA01 |
| Antipsychotics | Aripiprazole | N05AX12 |
| Antipsychotics | Chlorpromazine | N05AA01 |
| Antipsychotics | Clozapine | N05AH02 |
| Antipsychotics | Flupentixol | N05AF01 |
| Antipsychotics | Haloperidol | N05AD01 |
| Antipsychotics | Lithium | N05AN01 |
| Antipsychotics | Olanzapine | N05AH03 |
| Antipsychotics | Perphenazine | N05AB03 |
| Antipsychotics | Pimozide | N05AG02 |
| Antipsychotics | Quetiapine | N05AH04 |
| Antipsychotics | Risperidone | N05AX08 |
| Antipsychotics | Thioridazine | N05AC02 |
| Antipsychotics | Tiotixene | N05AF04 |
| Antipsychotics | Trifluoperazine | N05AB06 |
| Anxiolytics | Buspirone | N05BE01 |
| Anxiolytics | Hydroxyzine | N05BB01 |
| Benzodiazepine | Alprazolam | N05BA12 |
| Benzodiazepine | Chlordiazepoxide | N05BA02 |
| Benzodiazepine | Diazepam | N05BA01 |
| Benzodiazepine | Lorazepam | N05BA06 |
| Benzodiazepine | Oxazepam | N05BA04 |
| Illicit drugs | Cannabis | - |
| Illicit drugs | Cocaine | - |
| Illicit drugs | Crack Cocaine | - |
| Illicit drugs | Crystal Methamphetamine | - |
| Illicit drugs | Heroin | - |
| Illicit drugs | Kratom | - |
| Illicit drugs | MDMA (Ecstasy) | - |
| Illicit drugs | Opium | - |
| Opioids | Morphine | N02AA01 |
| Opioids | Oxycodone | N02AA05 |
| Opioids | Paracetamol+Codein | N02AJ06 |
| Opioids | Pethidine | N02AB02 |
| Opioids | Tramadol | N02AX02 |
| Other Nervous System Drugs | Betahistine | N07CA01 |
| Other Nervous System Drugs | Pyridostigmine | N07AA02 |
| Other Nervous System Drugs | Tetrabenazine | N07XX06 |
| Psychostimulants | Amfetamine | N06BA01 |
| Psychostimulants | Atomoxetine | N06BA09 |
| Psychostimulants | Caffeine | N06BC01 |
| Psychostimulants | Methylphenidate | N06BA04 |
| Psychostimulants | Piracetam | N06BX03 |
| Hypnotics and Sedatives | Chloral Hydrate | N05CC01 |
| Hypnotics and Sedatives | Flurazepam | N05CD01 |
| Hypnotics and Sedatives | Midazolam | N05CD08 |
| Hypnotics and Sedatives | Nitrazepam | N05CD02 |
| Hypnotics and Sedatives | Zolpidem | N05CF02 |
